# DOTA-Functionalized Polylysine: A High Number of DOTA Chelates Positively Influences the Biodistribution of Enzymatic Conjugated Anti-Tumor Antibody chCE7agl

**DOI:** 10.1371/journal.pone.0060350

**Published:** 2013-04-02

**Authors:** Jürgen Grünberg, Simone Jeger, Dikran Sarko, Patrick Dennler, Kurt Zimmermann, Walter Mier, Roger Schibli

**Affiliations:** 1 Center for Radiopharmaceutical Sciences ETH-PSI-USZ, Paul Scherrer Institute, Villigen, Switzerland; 2 Department of Nuclear Medicine, University Hospital Heidelberg, Heidelberg, Germany; Genentech, United States of America

## Abstract

Site-specific enzymatic reactions with microbial transglutaminase (mTGase) lead to a homogenous species of immunoconjugates with a defined ligand/antibody ratio. In the present study, we have investigated the influence of different numbers of 1,4,7,10-tetraazacyclododecane-N-N′-N′′-N′′′-tetraacetic acid (DOTA) chelats coupled to a decalysine backbone on the *in vivo* behavior of the chimeric monoclonal anti-L1CAM antibody chCE7agl. The enzymatic conjugation of (DOTA)_1_-decalysine, (DOTA)_3_-decalysine or (DOTA)_5_-decalysine to the antibody heavy chain (via Gln295/297) gave rise to immunoconjugates containing two, six or ten DOTA moieties respectively. Radiolabeling of the immunoconjugates with ^177^Lu yielded specific activities of approximately 70 MBq/mg, 400 MBq/mg and 700 MBq/mg with increasing numbers of DOTA chelates. Biodistribution experiments in SKOV3ip human ovarian cancer cell xenografts demonstrated a high and specific accumulation of radioactivity at the tumor site for all antibody derivatives with a maximal tumor accumulation of 43.6±4.3% ID/g at 24 h for chCE7agl-[(DOTA)-decalysine]_2_, 30.6±12.0% ID/g at 24 h for chCE7agl-[(DOTA)_3_-decalysine]_2_ and 49.9±3.1% ID/g at 48 h for chCE7agl-[(DOTA)_5_-decalysine)]_2_. The rapid elimination from the blood of chCE7agl-[(DOTA)-decalysine]_2_ (1.0±0.1% ID/g at 24 h) is associated with a high liver accumulation (23.2±4.6% ID/g at 24 h). This behavior changed depending on the numbers of DOTA moieties coupled to the decalysine peptide with a slower blood clearance (5.1±1.0 (DOTA)_3_ versus 11.7±1.4% ID/g (DOTA)_5_, p<0.005 at 24 h) and lower radioactivity levels in the liver (21.4±3.4 (DOTA)_3_ versus 5.8±0.7 (DOTA)_5_, p<0.005 at 24 h). We conclude that the site-specific and stoichiometric uniform conjugation of the highly DOTA-substituted decalysine ((DOTA)_5_-decalysine) to an anti-tumor antibody leads to the formation of immunoconjugates with high specific activity and excellent *in vivo* behavior and is a valuable option for radioimmunotherapy and potentially antibody-drug conjugates (ADCs).

## Introduction

One of the remaining challenges of immunoconjugation is product homogeneity with regard to site-specificity and stoichiometry of antibody modification [1]–[3]. Site-specifically conjugated tumor-targeting antibodies have been shown to exhibit a greater uptake at the cancerous site and less non-specific uptake in off-target tissues compared to heterogeneous immunoconjugates [4], [5]. A similar tendency has been observed for stoichiometry: antibodies with a high number of conjugated entities were cleared faster from the blood, counteracting the high tumor accumulation [6]. A possible explanation is that the body recognizes highly substituted immunoconjugates as a damaged form of the protein and quickly clears them from the blood [7]. Furthermore, it is common for randomly conjugated antibodies to be modified in positions that weaken or even abrogate antigen binding, which in turn decreases the efficacy of the targeting immunoconjugate [8]–[10]. It is therefore desirable to conjugate a moderate and defined number of entities per antibody molecule [6]. At the same time, it has to be taken into account that the lower the drug/antibody ratio, the more potent the drug needs to be [11]. In the case of radioimmunoconjugates (RICs), high specific activity is required in order to deliver therapeutic doses to the tumor site [12]. Using radiometal-labeled antibodies, this can be achieved by employing isotopes which are available with high specific activity, and also by functionalization of the protein with high numbers of metal chelating agents. However, it has been observed that chemically substituted radioimmunoconjugates with high numbers of metal chelators show higher uptake and retention in non-targeted organs and tissues while often clearing faster from the blood pool, resulting in poor target/non-target ratios [12]–[14].

To overcome this dilemma Slinkin *et al.* used an approach to attach a high number of chelating agents to the anticardiac myosin antibody R11D10 [15]. Upon conjugation of a polylysine chain containing on average ten deferoxamine molecules, they observed a loss of immunoreactivity of 4–5 fold for the immunoconjugate. Wängler *et al.* designed a dendrimer incorporating up to 128 chelating agents (DOTA) and conjugated it to the anti-EGFR antibody hMAb425 [16]. By comparing the novel antibody-dendrimer conjugates with conventionally produced immunoconjugates containing high numbers of single chelating molecules at multiple sites, they found that the number of conjugation sites had a dramatic effect on the immunoreactivity of the antibody, whereas dendrimer size did not influence the immunoreactivity of the derivatized antibody with comparable derivatization sites per molecule. In a recently published study, Ramli *et al.* have designed a tris-tetraazamacrocycle comprising three chelating moieties in order to increase the specific activity of a RIC [17]. In comparison to an analogous mono-tetraazamacrocycle, the tris-tetraazamacrocycle immunoconjugate showed higher specific activity upon radiolabeling with ^64^Cu. In an *in vivo* biodistribution study in tumor-bearing nude mice, both RICs showed similar tumor uptake. However, it is noteworthy that adverse liver uptake was threefold higher for the tris-tetraazamacrocycle RIC than for the mono-tetraazamacrocycle RIC. Furthermore, in the examples mentioned above, the exact sites of antibody modification were not known.

We have recently reported a novel strategy using an enzyme of the transglutaminase family (microbial transglutaminase, mTGase) for site-specific and stoichiometric uniform antibody modification with various lysine-comprising or lysine-mimicking substrates including transition metal chelators [4]. Transglutaminases form a stable isopeptidic bond between a lysine and a glutamine side chain with the loss of ammonia [18]. During our studies, we have identified Gln295 as the sole site of modification of different deglycosylated human IgG1-antibodies.

In this study, we make use of constructs comprising a decapeptide of lysine and different numbers of DOTA moieties attached to the lysine side chains. The oligolysine backbone was primarily chosen in order to permit a convergent synthesis with different numbers of DOTA moieties [19]. At the same time, the lysine residues enabled the recognition of the compounds by mTGase. This approach allowed the preparation of site-specifically modified, homogeneous immunoconjugates with increasingly well-defined chelator/mAb ratios. In this context we investigated the influence of the different numbers of DOTA chelats on the *in vitro* and *in vivo* characteristics of an antibody. We employed the anti-L1-CAM antibody chCE7agl (N297Q mutation), as it has shown excellent tumor-targeting capacity [20], [21].

## Materials and Methods

### General

The chCE7agl antibody was expressed and purified following a previously published protocol [21]. Microbial transglutaminase (from *Streptomyces mobaraensis*) was purchased from Zedira (Darmstadt, Germany). All chemicals were purchased from Sigma-Aldrich or Fluka, Buchs, Switzerland, unless otherwise stated. All cell culture media and additives were purchased from BioConcept (Allschwil, Switzerland). ^177^Lu chloride was purchased from ITM (Isotopen Technologien München) AG (Garching, Germany). 2-(1H-benzo-triazole-1-yl)-1,1,3,3-tetramethyluroniumhexafluorophosphate (HBTU) was purchased from Novabiochem (Schwalbach, Germany). The peptides were purified and analyzed by reversed-phase high-performance liquid chromatography (RP-HPLC) on a Chromolith SemiPrep 100 × 10 mm column (Merck, Germany), and characterized by LC/MS using an Exactive (Thermo Scientific) mass spectrometer. The composition of the immunoconjugates was determined via LC/MS analysis on a Waters LCT Premier mass spectrometer. Samples were chromatographed on an Uptisphere BP1 column (50 µm, 150 mm × 2 mm; Interchim, Montluçon, France) heated to 40°C, using a linear gradient starting from 20% A, 75% B and 5% C (solvent A, 0.1% formic acid in acetonitrile, solvent B, 0.1% formic acid in water, solvent C, isopropanol) and changing to 50% A, 45% B and 5% C in 25 min at a flow rate of 0.5 mL/min. The eluent was ionized using an electrospray source (ESI+). Data were collected with MassLynx V4.1 (Waters Corp., Millford, USA) and deconvolution was performed using MaxEnt1. Before LC/MS analysis, immunoconjugates (10 µg in 10 µL) were mixed with guanidine buffer (7.5 M guanidine·HCl, 0.1 M Tris·HCl, 1 mM EDTA, pH 7.4; 40 µL) and reacted with 50 mM DTT at 70°C for 30 min to reduce disulfide bonds and thereby separate the heavy and light chains of the antibody.

SKOV3ip1 cells were maintained in Dulbecco’s modified Eagle medium (DMEM; 4.5 g/L glucose) supplemented with 10% fetal calf serum (FCS), 2 mM of glutamine, 100 units/mL of penicillin, 100 µg/mL of streptomycin, and 0.25 µg/mL of fungizone. *In vivo* studies were performed in female athymic immunodeficient nude mice (CD1-foxn1^nu^; Charles River, Sulzfeld, Germany) in compliance with Swiss laws on animal protection.

### Preparation of Polylysine-DOTA Derivatives

Compounds were assembled on a 0.05 mmol Tenta Gel R Ram resin (Rapp Polymere, Germany) with a degree of substitution of 0.19 mmol/g. Fmoc L-lysine derivatives (Novabiochem, Schwalbach, Germany) with the following protecting groups were employed: Fmoc-Lys(Mtt)-OH, Fmoc-Lys(Boc)-OH, and Boc-Lys(Boc)-OH. The peptides were obtained by solid phase peptide synthesis (SPPS) in a fully automated peptide synthesizer (ABI 433A Applied Biosystems) using fluorenylmethyloxycarbonyl (Fmoc) chemistry. HBTU was used as the coupling agent. In order to attach DOTA to the N^ε^ side chain of specific lysines, Fmoc-Lys(Mtt)-OH was used in the positions to be modified. The N^ε^ Mtt protecting groups were removed using 1% trifluoroacetic acid (TFA) in dichloromethane (DCM), tris-^t^Bu-DOTA (tris-^t^Bu-1,4,7,10-tetraazacyclododecane-N-N′-N′′-N′′′-tetraacetic acid) was coupled to obtain DOTA-conjugated peptides.

### Enymatic Conjugation of chCE7degl and chCE7agl

Deglycosylation of mAb chCE7 was performed as previously described [4]. For enzymatic conjugation, chCE7degl and chCE7agl were dissolved in 0.04 M Tris-HCl buffer (pH 7.0) and potassium-free PBS buffer (pH 9.0) to a concentration of 1 mg/mL, respectively. An antibody/ligand molar ratio of 1/80 was used and mTGase (specific activity: 31 U/mg) was added at an E/S ratio of 1/3.5 (chCE7degl) and 1/6 (chCE7agl) (w/w). The reaction mixture was incubated for 16 h at 37°C with gentle agitation. The completeness of the reaction was monitored by LC/MS analysis as described above. Before radiolabeling, centrifugation-dialysis was performed (Vivaspin MWCO 50 kDa, Vivascience, Winkel, Switzerland) in 0.25 M ammonium acetate buffer (pH 5.5). This step also removed both the excess of chelator substrates and the enzyme. The final concentration of the antibody conjugates was ∼1.5 mg/mL.

### Radiolabeling

For small scale radiolabeling, 50 µg of the immunoconjugate was reacted with ^177^Lu solution (5 µL with 70 MBq) in 225 µL ammonium acetate buffer (0.25 M, pH 5.5) at 37°C for 2.5 h. After incubation, 25 µL of a 50 mM EDTA solution was added to give a final concentration of 5 mM EDTA and stirred for 5 min to complex free lutetium. The radioimmunoconjugates were analyzed and purified using size exclusion chromatography (SEC) on a Superose-12 Fast Protein Liquid Chromatography (FPLC) column (GE Healthcare, Dübendorf, Switzerland) and eluted with PBS buffer containing 1 M NaCl at a flow rate of 0.5 mL/min. Fractions of 500 µL were collected and the major peak fractions were pooled, after which specific activities were determined. For the animal study the radiolabeling procedure was scaled up and 500–1000 µg antibody derivatives were labeled with 500–700 MBq ^177^Lu in a volume of 900 µL ammonium acetate buffer.

### Quality Control of Radiolabeled Purified Preparations

The immunoreactivities of the ^177^Lu-labeled derivatives were assayed with cell-binding tests using increasing numbers of SKOV3ip1 cells as described before [22]. Briefly, triplicate samples of a fixed tracer amount of the radioimmunoconjugate (50 ng) were incubated with increasing numbers of cells (0.063–2 × 10^6^) in a total volume of 550 µL PBS on a shaking platform at 37°C for 2 h. Non-specific binding was determined by parallel incubations in the presence of 10 µg of the unconjugated antibody. Human plasma stability studies were performed according to a previously published protocol [23]. Briefly, 750 µL of human plasma was incubated with 75 µL of radioimmunoconjugate (100–500 kBq, 1–5 µg) at 37°C for up to 120 h. Samples of 200 µL were taken immediately after addition (controls) at 24, 48, and 120 h and EDTA was added to give a final concentration of 5 mM in order to complex free radionuclides. Samples were analyzed by FPLC size exclusion chromatography on a Superose-12 column and a gamma measuring cell.

### Biodistribution Studies

#### Ethics statement

Animal studies were conducted in compliance with the Swiss laws on animal protection. All experiments were approved by the Tierversuchskommission der Kantone BS-BL-AG, Switzerland, and permitted by the local government (Departement Gesundheit und Soziales, Veterinärdienst des Kantons Aargau, Switzerland; permission number: 75528). All efforts were made to minimize suffering. Housing and animal husbandry was conducted according to local law on animal protection.

#### Execution

Female CD1-foxn1^nu^ mice, 5 weeks old, with a weight of 16–19 g (Charles River, Sulzfeld, Germany) were injected in the peritoneum (i.p.) with 5 x 10^6^ SKOV3ip1 human ovarian carcinoma cells. The cells were a gift from Prof. P. Altevogt (German Cancer Center, Heidelberg, Germany) and the identity of the cell line was determined by STR profiling (IdentiCell, Aarhus, Denmark). After three weeks, groups of 15–16 animals were injected i.p. with 1.2 MBq of chCE7agl-[(DOTA)-decalysine]_2_, chCE7agl-[(DOTA)_3_-decalysine]_2_ or 0.9 MBq of chCE7agl-[(DOTA)_5_-decalysine]_2_ (400 µl PBS containing 12.4 µg protein). At 24, 48, 72, and 120 h post injection, groups of 3–4 mice for each derivative were sacrificed. Tumors and major organs were weighed and measured together with an aliquot of the injected solution in a gamma counter. Results are expressed as % injected dose per gram (%ID/g). Statistical analysis of data was performed using the Student’s *t* test (unpaired, two-tailed).

### Isoelectric Focusing

Isoelectric focusing (IEF) was conducted on an IsoGel IEF agarose plate (100 mm × 125 mm; pH 3–10) from Lonza (Rockwill, ME, USA) according to manufacturers instruction. Samples of chCE7agl-[(DOTA)_n_-decalysine]_2_ and chCE7agl (5 µg/µL) were dialyzed against deionized water, after which 5 µL of each sample was loaded 4 cm from the cathode into an applicator mask slot. IEF protein marker mixture from Serva, pI 3.5–10.7 (Serva, Heidelberg, Germany) or Bio-Rad, pI 4.45–9.6 (Bio-Rad Laboratories GmbH, München, Germany) was used to determine the pI of the derivatives. Focusing was performed with a Multiphor II electrophoresis unit (GE Healthcare, Dübendorf, Switzerland). After prefocusing at 1 W constant power for 10 min and focusing (20 W power limit; 1000 V voltage limit; 90 min; the current dropped at the end of the run to a steady state of approximately 3 mA) the gel was fixed in 30% trichloroacetic acid for 20 min. After drying, proteins were stained with a standard Coomassie stain solution.

## Results and Discussion

### Synthesis of (DOTA)_n_-decalysine

**Figure 1:**
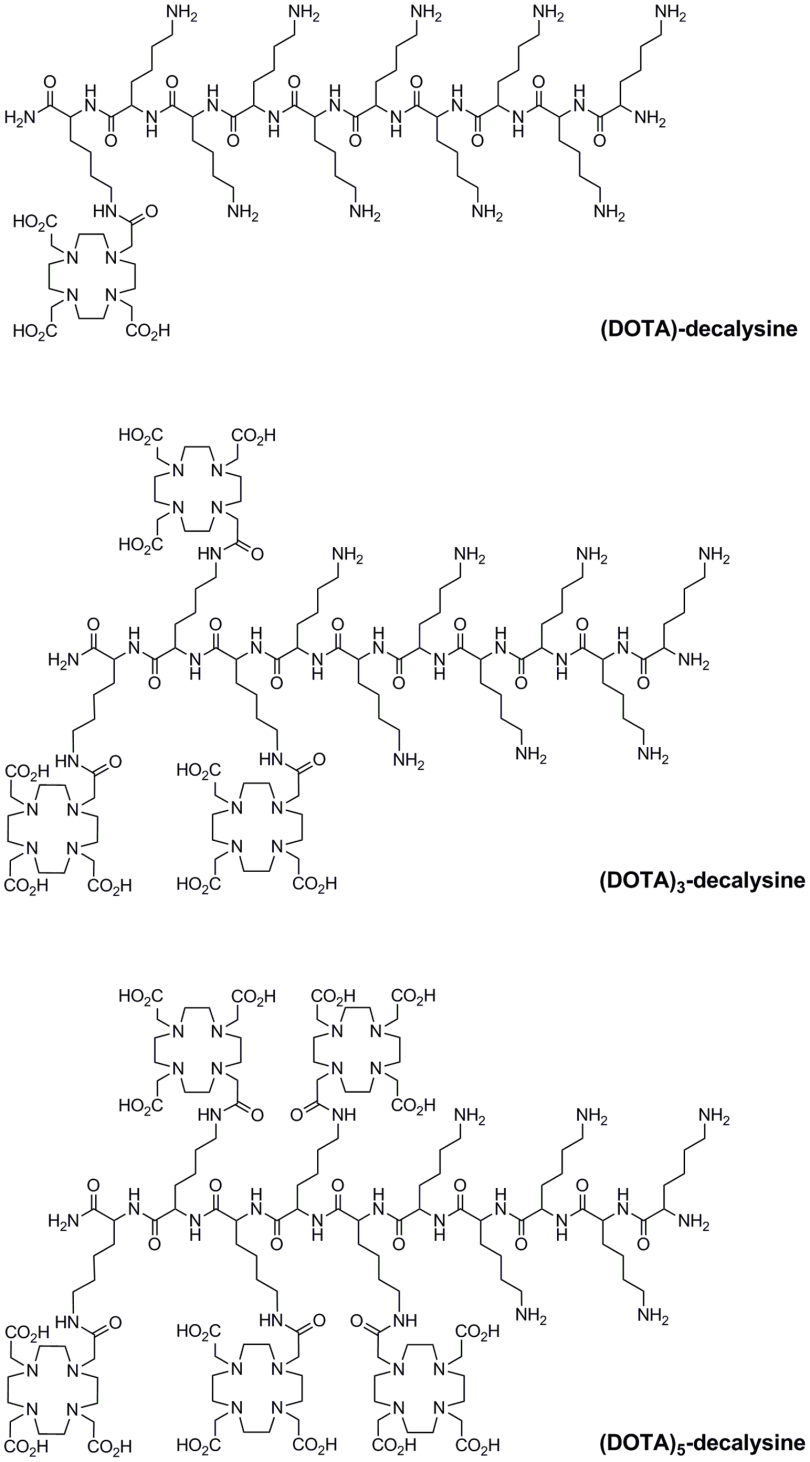
Structures of (DOTA)-decalysine, (DOTA)_3_-decalysine, and (DOTA)_5_-decalysine.

The structures of (DOTA)-decalysine, (DOTA)_3_-decalysine and (DOTA)_5_-decalysine are depicted in Figure 1. Fmoc-Lys(Mtt)-OH was used on the DOTA-modified positions, Fmoc-Lys(Boc)-OH was used on the unmodified positions, while Boc-Lys(Boc)-OH was used as the N-terminal amino acid. Fmoc-Lys(Mtt)-OH residue was used at the C-terminal residue (position 10), positions 8–10, and positions 6–10 of (DOTA)-decalysine, (DOTA)_3_-decalysine and (DOTA)_5_-decalysine respectively, to attach DOTA to the N^ε^ side chains. The cleavage of Mtt was repeated until monitoring of the disappearance of the yellow color of the trityl cation indicated an almost complete reaction. Overall yields of (DOTA)_3_-decalysine and (DOTA)_5_-decalysine were 30–40%. Apparently, a small fraction of the Boc protecting groups was cleaved during the prolonged exposure to the Mtt cleavage solution.

The structure of the (DOTA)_n_-decalysine derivatives has two inherent advantages: a) the oligolysine backbone permitted the convergent synthesis with different numbers of DOTA moieties [19], and b) the lysine side chains are recognized by the mTGase as a potential substrate.

### Antibody Modification

Human IgG1-antibodies are generally *N*-glycosylated at position Asn297, which is located in the Fc part of the protein [24]. We realized during our previous studies that the glycosylated IgGs are not or only marginally modified via glutamine side chains with corresponding substrates using mTGase (or other transglutaminases) [4], [25]. It appears that in most of the tested IgGs, there were no accessible glutamines with the correct steric environment [26], including preferred neighboring amino acids [27] or structural flexibility [26], which could have been modified with a lysine or lysine-mimicking substrate using mTGase. However, it became apparent that deglycosylated or aglycosylated variants of IgGs are readily modified with a defined number of substrates depending on the number of accessible glutamine residues in this flexible region where glycosylation normally occurs [28]. We have demonstrated that deglycosylated IgGs (including the anti-L1-CAM antibody chCE7 and the anti-CD20 antibody rituximab) can be modified with one chemical entity per heavy chain via Gln295 using mTGase [4]. Moreover, the aglycosylated variant of chCE7 (chCE7agl, with an Asn297Gln mutation) can be readily modified with two lysine substrates per HC at the neighboring glutamine residues Gln295 and Gln297. Being able to predict the exact site of modification and the stoichiometry, we were interested in systematically investigating the influence of increasing, even numbers of DOTA chelators on the radiolabeling yield (specific activity) as well as the *in vivo* behavior using (DOTA)_n_-decalysine derivatives (n = 1, 3, 5). Modification of lysine residues with glutamine substrates was difficult because of premature, irreversible, enzymatic hydrolysis of the corresponding glutamine substrates to glutamate. Recently, Spolaore *et al.* [29] showed in an elegant study that site-specific protein modification by transglutaminase requires the glutamine and lysine residues to be either in a flexible or a locally unfolded region of the protein. This could also explain why the site-specific derivatization of antibody chCE7agl by mTGase with a Gln substrate could not be accomplished as there is not a lysine residue embedded in such a flexible or unfolded structure.

**Figure 2:**
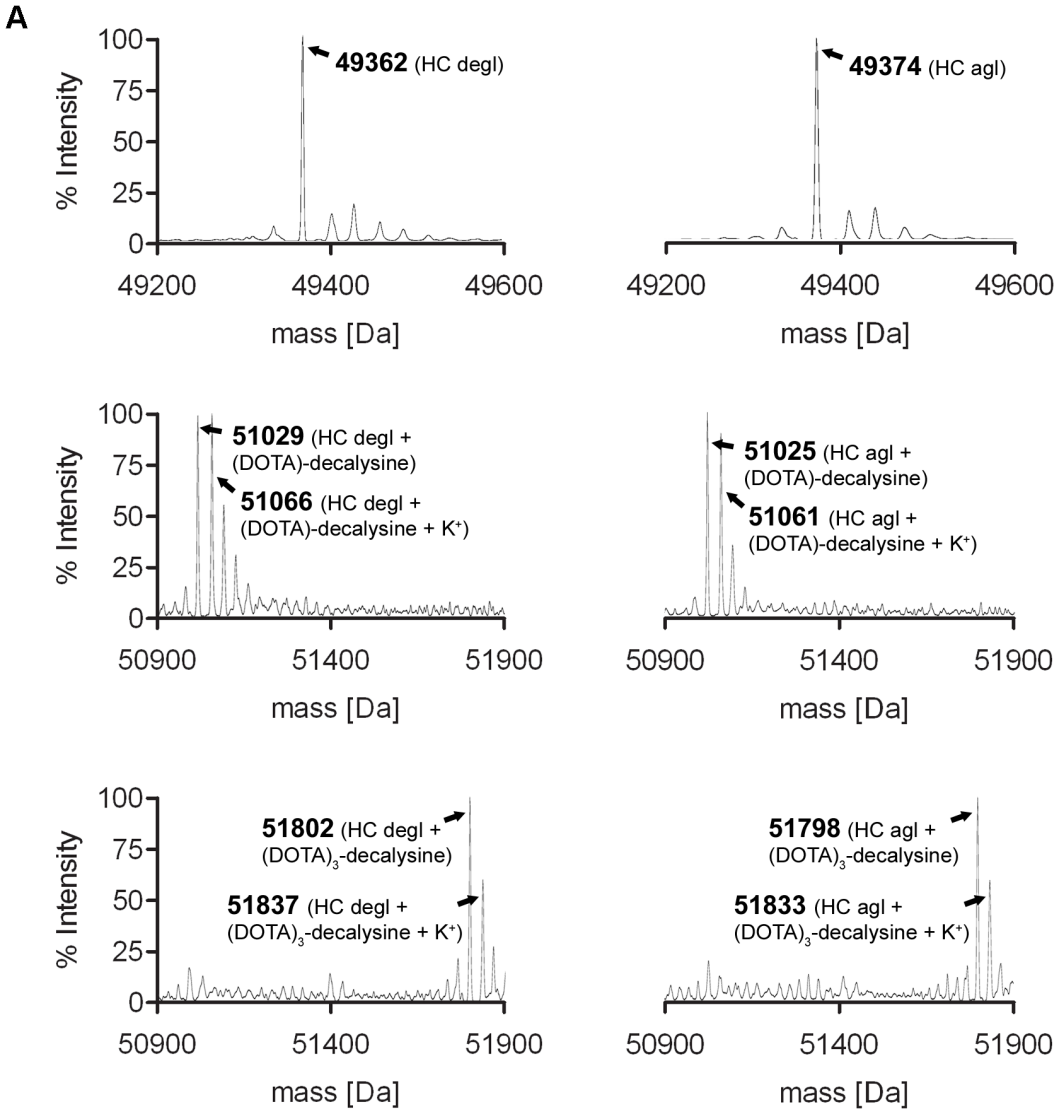
LC-ESI-TOF mass spectrometry analysis of the heavy chain (HC) of the used antibodies. Deconvolution of the raw data was accomplished by MaxEnt1. **A.** Mass spectrum of HC for chCE7degl (left) and chCE7agl (right). **B.** Mass spectrum of HC for chCE7degl coupled to (DOTA)-decalysine (left) and chCE7agl coupled to (DOTA)-decalysine (right); **C.** Mass spectrum of HC for chCE7degl coupled to (DOTA)_3_-decalysine (left) and chCE7agl coupled to (DOTA)_3_-decalysine (right).

**Figure 3:**
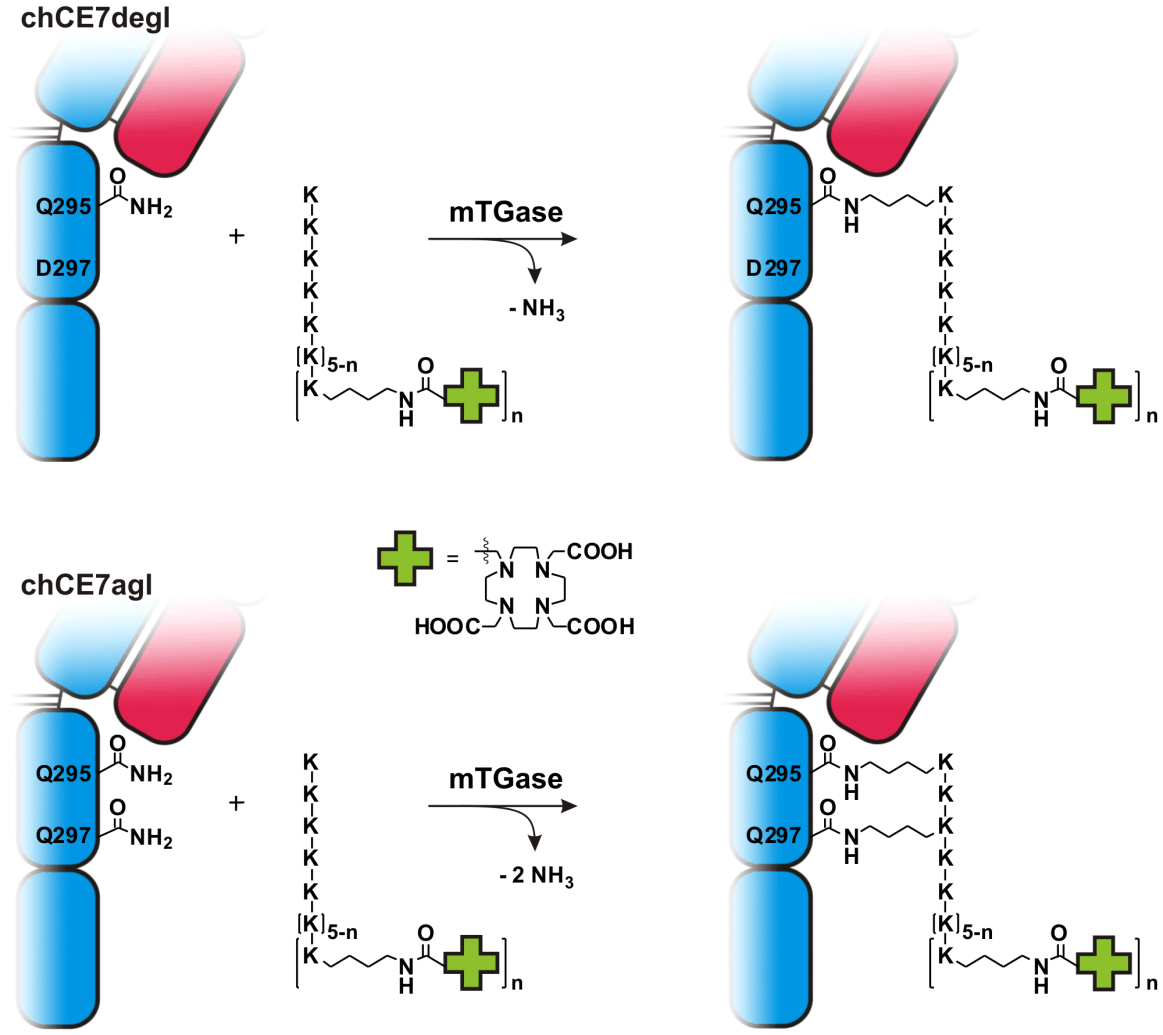
Enzymatic conjugation of (DOTA)_n_-decalysine to chCE7degl or chCE7agl, an aglycosylated variant of the antibody. Although there are two enzymatic recognition sites per heavy chain of chCE7agl (Gln295 and Gln297), only one molecule of (DOTA)_n_-decalysine (n = 1, 3, 5) was conjugated using transglutaminase. Interestingly, two isopeptide bonds were formed between one substrate and one heavy chain, as indicated by the loss of two ammonia molecules (bottom).

We enzymatically conjugated (DOTA)_n_-decalysine to chCE7degl and chCE7agl by using an antibody/ligand ration of 1/80. The mixture was incubated for 16 h at 37°C, after which MS analyses confirmed in all cases the formation of single species of immunoconjugates with a defined antibody/ligand ratio (Figure 2, Table 1). The mass peak of the heavy chain (HC) of the deglycosylated antibody chCE7degl is 49.362 kDa (Figure 2A, left). The HC of the immunoconjugates revealed masses at 51.029 kDa (chCE7degl-(DOTA)-decalysine; Figure 2B, left) and 51.802 kDa (chCE7degl-(DOTA)_3_-decalysine; Figure 2C, left). The masses indicate the loss of one molecule of NH_3_ as expected due to the formation of the isopeptidic bond (Figure 3). The mass of the chCE7agl heavy chain is 49.374 kDa (Figure 2A, right). The masses of the conjugates were found to be 51.025 kDa for HC of chCE7agl-(DOTA)-decalysine (Figure 2B, right), 51.798 kDa for HC of chCE7agl-(DOTA)_3_-decalysine (Figure 2C, right). As evident from the MS spectra, the major MS signal was accompanied by satellite peaks with a mass difference of 35–37 Da. We attribute this signal to incorporation of K^+^ ions into the DOTA chelate. All data are summarized in Table 1. MS spectra of the chCE7degl- and chCE7agl-(DOTA)_5_-conjugate were difficult to deconvolute since the signal to background ratio is smaller than for (DOTA)-decalysine and (DOTA)_3_-decalysine conjugates. However, we observed the desired final product with the expected mass shift of 3212 Da and 3195 Da for chCE7degl and chCE7agl, respectively (see Supporting Information S1). Analysis of the antibody light chain of all immunoconjugates revealed no shift in the molecular mass compared to the reference (data not shown). We did not observe significant differences between the three substrates with respect to reaction kinetics. However, in the course of the study we also tested (DOTA)_6_-decalysine (data not shown). In this case, we observed only partial functionalization of the HC even after extended reaction time or further addition of enzyme. It seems that (DOTA)_6_-decalysine is a much poorer substrate for the enzyme, presumably due to steric influences and the negatively-charged DOTA close to the free lysine side chains.

**Table 1:** Mass spectrometry results of the unmodified and modified heavy chains (HC).

Antibody	Ligand	Calculated Mass [Da]	Observed Mass [Da]
chCE7degl	No ligand	49362	49362
	(DOTA)-decalysine (1684 Da)	51029	51029
	(DOTA)-decalysine+Potassium	51066	51066
	(DOTA)_3_-decalysine (2457 Da)	51802	51802
	(DOTA)_3_-decalysine+Potassium	51839	51837
chCE7agl	No ligand	49374	49374
	(DOTA)-decalysine (1684 Da)	51024	51025
	(DOTA)-decalysine+Potassium	51061	51061
	(DOTA)_3_-decalysine (2457 Da)	51797	51798
	(DOTA)_3_-decalysine+Potassium	51834	51833

Surprisingly, we observed for all of the (DOTA)_n_-decalysines conjugates of chCE7agl only one molecule of (DOTA)-decalysine (MW: 1684 Da), (DOTA)_3_-decalysine (MW: 2457 Da), and (DOTA)_5_-decalysine (MW: 3229 Da) per HC (Figure 2, Table 1, Supporting Information S1) and not two as we had expected based on our previous findings [4]. The mass of the functionalized HC revealed that the enzymatic conjugation was accompanied by the loss of two ammonia molecules (2 × 17 Da), indicating the formation of two isopeptide bonds per substrate with the heavy chain. This can only be explained if two of the lysine residues of (DOTA)-, (DOTA)_3_-, and (DOTA)_5_-decalysine are conjugated to the neighboring glutamine residues Gln295 and Gln297 of antibody chCE7agl (Figure 3).

### Radiolabeling and *in vitro* Characterization of chCE7agl-[(DOTA)_n_-decalysine]_2_ Derivatives

To assess the radiolabeling efficiency, 50 µg of antibody derivatives were labeled with 70 MBq of ^177^Lu in 225 µL acetate buffer pH 5.5 for 2.5 h at 37°C. Under these conditions we reached specific activities of approximately 70 MBq/mg, 400 MBq/mg and 700 MBq/mg for chCE7agl-[(DOTA)-, chCE7agl-[(DOTA)_3_- and chCE7agl-[(DOTA)_5_-decalysine]_2_, respectively (Table 2). Thus, increasing numbers of DOTA moities coupled to the decalysine substrate was accompanied with an increasing specific activity of the immunoconjugates. These specific activities are in line with specific activities we observed for mAb ^177^Lu-chCE7agl with an average of 4.2 DOTA-ligands chemically coupled to the antibody [20]. As the specific activity of this particular antibody was between 52–133 MBq/mg protein, it is possible to reach much higher specific activities with the enzymatic modified mAbs in combination with DOTA-decalysine derivatives. It should be noted that the radiolabeling procedure was not further optimized since this was not the subject of the current study. However, higher specific activities can potentially be reached.

**Table 2:** General *in vitro* and *in vivo* features of chCE7agl-[(DOTA)_n_-decalysine]_2_ (n = 1, 3, 5).

	chCE7agl-[(DOTA)-decalysine]_2_	chCE7agl-[(DOTA)_3_-decalysine]_2_	chCE7agl-[(DOTA)_5_-decalysine]_2_
Total number of DOTA	2	6	10
Specific activity	Ca. 70 MBq/mg	Ca. 400 MBq/mg	Ca. 700 MBq/mg
pI	>9.5	>9.5	7.8–8.3
Immunoreactivity	81.3%	51.0%	39.2%
Tumor-to-blood*	70.2±24.6	15.3±2.2	7.9±0.0
Tumor-to-Liver*	1.7±0.9	2.2±0.6	8.6±0.8
Liver-to-blood*	43.0±7.6	7.2±1.2	0.93±0.1

* 72 h p.i.

We tested the stability of the chCE7agl-[(DOTA)_n_-decalysine]_2_ derivatives in human plasma. All immunoconjugates remained stable in human plasma for at least 120 h (>98%) and the immunoreactivity as determined by cell binding assays was between 39–81% (Table 2). Fischer *et al.* have reported immunoreactivities of between 62–87% for a chemically modified ^177^Lu-DOTA-chCE7agl with an average content of 4.2 DOTA/mAb as determined by Lindmo assays [20]. It is not clear why increasing numbers of DOTA moieties leads to a reduced immunoreactivity of our conjugates. Since the site of modification is constant for all derivatives and distant from the antibody binding site, we can exclude steric hindrance. The cell binding assays were not conducted immediately after labeling and so we cannot exclude radiolytic damage of the antibodies with high specific activities. This could explain the loss of immunoreactivity of the RICs. Although we have not tested this we propose the use of ascorbic or gentisic acid to prevent radiolysis. Nevertheless, the chCE7agl-[(DOTA)_5_-decalysine]_2_ retained the capacity for specific delivery of ^177^Lu to the tumor site *in vivo* (Table 3).

**Table 3:** Biodistribution of ^177^Lu-labeled chCE7agl-[(DOTA)_n_-decalysine]_2_ in nude mice with human ovarian cancer (SKOV3ip) metastases.

^177^Lu-chCE7agl-[(DOTA)-decalysine]_2_				
Organ(%ID/g)	24 h	48 h	72 h	120 h
Tumor	43.6±4.3	34.3±9.0	27.6±10.0	17.6±7.8
Blood	1.0±0.1	0.7±0.1	0.4±0.1	0.2±0.1
Liver	23.2±4.6	19.1±4.1	16.9±3.4	6.9±1.1
Spleen	13.0±4.7	10.4±4.6	8.6±1.5	4.5±1.4
Kidney	8.9±2.0	8.7±1.6	7.3±1.5	4.8±1.1
Heart	1.0±0.2	0.8±0.1	0.6±0.1	0.3±0.1
Stomach	3.2±1.2	3.4±1.6	3.1±2.2	1.0±0.6
Intestine	2.0±0.6	2.3±0.9	2.0±0.3	0.8±0.2
Muscle	0.6±0.1	0.7±0.2	0.5±0.1	0.3±0.1
Bone	1.7±0.3	1.3±0.1	0.9±0.2	0.6±0.1
**^177^Lu-chCE7agl-[(DOTA)_3_-decalysine]_2_**				
**Organ** **(%ID/g)**	**24 h**	**48 h**	**72 h**	**120 h**
Tumor	30.6±12.0	25.0±5.0	26.8±3.1	16.0±4.7
Blood	5.1±1.0	2.5±0.3	1.2±0.1	1.0±0.3
Liver	21.4±3.4	14.1±2.6	12.8±2.5	6.7±1.3
Spleen	11.5±3.4	14.1±2.6	12.8±2.5	6.7±1.3
Kidney	11.7±1.7	10.8±1.9	9.6±1.4	7.1±1.1
Heart	2.4±0.3	1.4±0.2	1.1±0.1	0.7±0.2
Stomach	1.8±0.2	2.6±1.6	1.2±0.4	0.7±0.2
Intestine	2.4±0.5	1.8±0.6	1.3±0.3	0.9±0.2
Muscle	1.5±0.6	1.0±0.1	0.5±0.3	0.3±0.1
Bone	2.3±0.5	1.8±0.4	1.1±0.1	0.9±0.3
**^177^Lu-chCE7agl-[(DOTA)_5_-decalysine]_2_**				
**Organ** **(%ID/g)**	**24 h**	**48 h**	**72 h**	**120 h**
Tumor	41.1±6.9	49.9±3.1	42.4±3.8	33.3±14.0
Blood	11.8±1.4	10.2±0.8	5.3±0.2	2.0±1.1
Liver	5.8±0.7	5.7±0.5	5.0±0.8	6.1±0.5
Spleen	7.3±2.3	5.7±0.7	8.1±1.3	5.5±0.7
Kidney	6.2±0.5	6.7±0.9	6.3±0.5	4.5±1.2
Heart	4.5±0.8	3.8±0.7	2.3±0.3	0.9±0.7
Stomach	1.7±0.9	1.9±1.3	1.3±0.5	0.8±0.6
Intestine	1.9±0.2	1.9±0.5	1.3±0.3	0.9±0.3
Muscle	1.5±0.1	1.2±0.1	0.7±0.5	0.3±0.2
Bone	2.1±0.4	2.0±0.1	2.1±1.1	1.1±0.1

**Figure 4:**
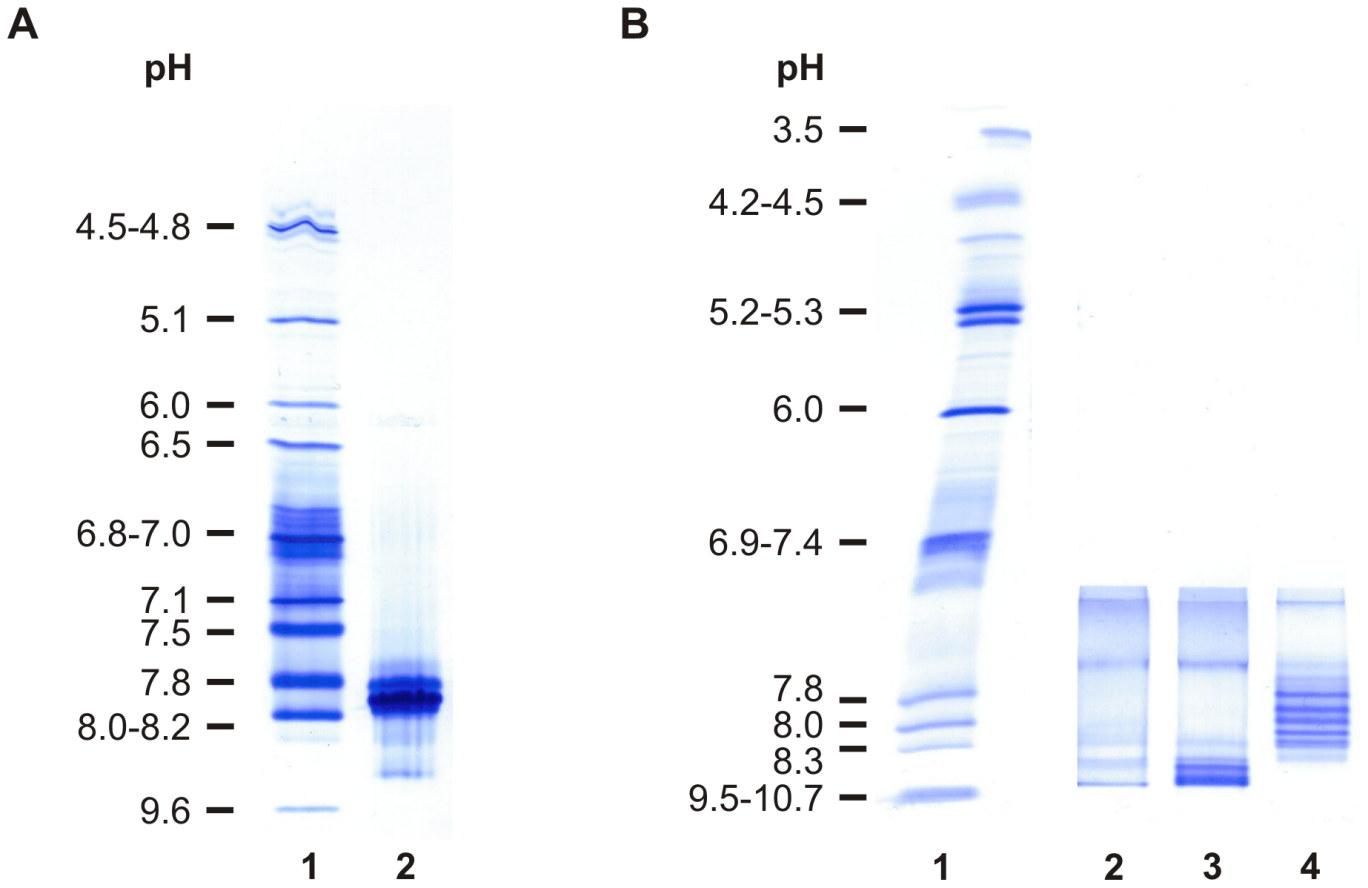
Isoelectric focusing of chCE7agl and chCE7agl-[(DOTA)_n_-decalysine]_2_ on an IEF agarose plate pH 3–10. Samples with a concentration of 5 µg/µL were dialyzed against deionized water before loading and 5 µL of each probe were loaded 4 cm from the cathode. After prefocusing at 1 W constant power for 10 min and focusing for 90 min (20 W power limit; 1000 voltage limit) the gels were fixed in 30% TCA and proteins were stained with Coomassie. **A.** Lane 1: IEF standard Biorad No 161-0310; lane 2: The parent antibody chCE7agl revealed a pI of 7.8. **B.** Lane 1: IEF marker Serva No 39212.01. The (DOTA)_n_-decalysine conjugates showing anodal movement depending on the numbers of DOTA chelats coupled to the decalysine. Lane 2: chCE7agl-[(DOTA)-decalysine]_2_; lane 3: chCE7agl-[(DOTA)_3_-decalysine]_2_; lane 4: chCE7agl-[(DOTA)_5_-decalysine]_2_.

Shifts in isoelectric point (pI) can produce changes in the biodistribution and kinetics of immunoconjugates. In general, an increase in net positive charge generally results in increased tissue retention and increased blood clearance [30]. Slinkin *et al.* observed large differences in biodistribution among anti-CEA F(ab’)_2_ fragments conjugated to chelating polymers with various charges [31]. While the most positively charged conjugate was immediately trapped in liver and kidneys, a slightly negative counterpart exhibited more favorable *in vivo* behavior. Therefore, we determined the isoelectric point of chCE7agl and the different chCE7agl-[(DOTA)_n_-decalysine]_2_ conjugates on an IsoGel IEF agarose plate (pH 3–10). The pI of the unmodified chCE7agl antibody was ∼ 7.8. Two species of the mAb are visible (Figure 4A, lane 2). chCE7agl-[(DOTA)-decalysine]_2_ (Figure 4B, lane 2) and chCE7-[(DOTA)_3_-decalysine]_2_ (Figure 4B, lane 3) were >9.5, thus rather more positively charged. The chCE7agl-[(DOTA)_5_-decalysine]_2_ (Figure 4B, lane 4) revealed a pI of between 7.8–8.3, which is much closer to the pI of the unmodified mAb. Isoelectric points of human IgGs are found to range from 6.4 to 9.0 [32]. Thus both immunocojugates with low DOTA substitutions had an unphysiologically high pI of greater than 9.5. The multiple bands of the antibody conjugates most likely represents the different protonation of the DOTA chelate. This would be consistent with the observation that the number of bands increases with the number of DOTA chelates coupled to the decalysine ligand. The immunoconjugates were always verified by LC/MS and purified after enzymatic conjugation. Therefore all conjugates loaded on the IEF-gel represented a homogenous species with a defined ligand/antibody ratio and thus, a defined number of DOTA chelates. We can exclude radiolysis of the conjugates, because unlabeled immunoconjugates were used for the determination of the pI.

### Biodistribution Studies of Lu-177 Labeled chCE7agl-[(DOTA)_n_-decalysine]_2_ Derivatives

The results of the time-dependent biodistribution data are summarized in Table 3. The immunoconjugates reached a maximal tumor accumulation at 24 h with 43.6±4.3% ID/g and 30.6±12.0% ID/g for chCE7agl-[(DOTA)-decalysine]_2_ and chCE7agl-[(DOTA)_3_-decalysine]_2_ respectively. From this point the accumulated activity declines to 34.3±9.0 (48 h) and 17.6±7.8% ID/g (120 h) for chCE7agl-[(DOTA)-decalysine]_2_. The same trend applied for chCE7agl-[(DOTA)_3_-decalysine]_2_. For the two conjugates, the blood pool radioactivity dropped to 1.0±0.1 chCE7agl-[(DOTA)-decalysine]_2_ and 5.1±0.15% ID/g chCE7agl-[(DOTA)_3_-decalysine]_2_ at 24 h. At the same time high liver accumulations of over 20% ID/g were observed. We speculate that the high tumor accumulation of these two derivatives at 24 h was due to the injection route of the conjugates. Otherwise, more of the conjugates may be trapped in the liver. The situation was different for chCE7agl-[(DOTA)_5_-decalysine]_2_: this conjugate showed a maximal tumor uptake at 48 h p.i. with 49.9±3.1% ID/g (p<0.05; 1 vs. 5 and p<0.005; 3 vs. 5) with a slow drop in uptake at 72 h (42.4±3.8%, p≤0.05) and 120 h p.i. (33.3±14.1% ID/g, p<0.005). This higher tumor accumulation was associated with a higher blood pool concentration (11.8±1.4–2.0±1.1% ID/g 24–122 h) of the conjugate over the whole time period (p<0.005; 1 and 3 vs. 5). Furthermore, the high tumor accumulation persisted in the same way as has been described for ^177^Lu-DOTA chCE7agl [20]. We assumed that this high and persistent tumor accumulation is due to the longer half-life of the immunoconjugate in the blood pool. The liver uptake for chCE7agl-[(DOTA)_5_-decalysine]_2_ was only 5.8% ID/g at 24 h (p<0.005). Thus, our results reflect to a certain extent the pI-dependence of biodistribution of radioimmunoconjugates reported in the literature.

The differences in blood clearance and tumor uptake of the three tested radioimmunoconjugates resulted in significantly different tumor-to-organ ratios. ^177^Lu-chCE7agl-[(DOTA)-decalysine]_2_ revealed high tumor-to-blood (between 44.0±6.5 at 24 h to 87.8±33.0 at 120 h) and liver-to-blood (between 23.2±3.6 at 24 h to 36.4±10.6 at 120 h) ratios at all time points (in Table 2 the values are shown for 72 h). The higher values are both a consequence of faster blood clearance and higher liver accumulation. ^177^Lu-chCE7agl-[(DOTA)_3_-decalysine]_2_ and ^177^Lu-chCE7agl-[(DOTA)_5_-decalysine]_2_ showed similar tumor-to blood ratios (between 6.1±2.1/3.5±0.7 at 24 h to 15.9±2.7/17.8±2.3 at 120 h), while, ^177^Lu-chCE7agl-[(DOTA)_5_-decalysine]_2_ displayed significantly better tumor-to-liver ratios (between 7.1±1.0 at 24 h to 5.6±2.9 at 120 h) than the conjugates with lower amounts of DOTA ligands. This is somewhat contrary to the trend observed by Knogler *et al.* where the chCE7agl was chemically functionalized with 7–15 DOTA chelators (randomly attached to lysine side chains of the mAb) [14]. They observed liver-to-blood ratios of 17.9±3.4 with an average of 15 chelators/mAb and ratios <1 with a lower number of chelators (this study has been performed in non-tumor bearing CD1-foxn1^nu^ mice), while Kukis *et al.* observed a similar trend with a murine anti-lymphoma IgG2a mAb Lym-1 chemically functionalized with different numbers (an average of 2 to 11 chelators/mAb) of the macrocyclic chelating agent TETA (1,4,8,11-tetraazacyclotetradecane-1,4,8,11-tetraacetic acid) labeled with ^67^Cu. In these studies, higher number of chelators resulted in lower tumor uptake, faster blood clearance and higher liver accumulation [33].

Recently, Fischer *et al.* [20] published a study using chemically modified ^177^Lu-DOTA-chCE7agl with an average number of 4.2 DOTA/mAb (0.3 or 1.8 on light and heavy chain, respectively) and a range of 1 to 9 ligands/antibody molecule. These mAbs revealed an excellent biodistribution with high and persistent tumor accumulation and low activity in off-target organs. This antibody was highly potent in the treatment of disseminated ovarian tumor nodules *in vivo*. The chemically modified mAbs described by Fischer *et al*. [20] were injected intravenously whereas the enzymatic conjugated mAbs in this study were injected intraperitoneally. This precludes a direct comparison of the biodistribution results. However, *in vivo* bioditribution of the chCE7agl-[(DOTA)_5_-decalysine]_2_ conjugate was very similar to the described chemically modified mAbs. Importantly, the enzymatic conjugations lead to immunoconjugates with a uniform and well-defined substitution only on the heavy chain. For a possible subsequent approval by the authorities, this can be advantageous.

### Conclusions

We have demonstrated that it is possible to use microbial transglutaminase to site-specifically modify the chimeric IgG1-type tumor-targeting anti-L1CAM antibody chCE7agl with various numbers of DOTA metal chelating systems attached to a polylysine backbone. This method allowed the production of homogenous immunoconjugate with a precise and predictable number of metal chelating agents per antibody. The site of modification was identified to be Gln295 and Gln297, which are located in a flexible loop region of the Fc-part of the antibody chCE7. Increasing numbers of DOTA moieties coupled to the decalysine substrate was accompanied by an increasing specific activity of the immunoconjugates when labeled with ^177^LuCl_3_. The advantage of the high specific activity was not counteracted by the simultaneous decrease of the immunoreactivity. In mice bearing human SKOV3ip1 tumor xenografts expressing the antigen L1CAM all immunoconjugates showed high tumor uptake at 24 h after intraperitoneal administration. However, high liver uptake and fast clearance from the blood pool was observed for the immunoconjugates with higher isoelectric points (pI >9.5). Therefore, our results provide evidence that ligand/mAb ratios *per se* are not the sole factor which influence target-to-organ ratios of (radio)immunoconjugates but factors such as pI and hydrophilicity/lipophilicity (influenced by the spacer entities, for example) are equally important. While this might not be surprising, the possibility of site-specific and stoichiometric functionalization of targeted mAb via our enzymatic method permits a systematic investigation of the influence of the individual factors in order to optimize the pharmacological properties of the radioimmunoconjugates.

## Supporting Information

Supporting Information S1 (DOC)
