# Supplementary material for: DOTA-Functionalized Polylysine: A High Number of DOTA Chelates Positively Influences the Biodistribution of Enzymatic Conjugated Anti-Tumor Antibody chCE7agl
Source: PLoS One. 2013 Apr 2;8(4):e60350. doi: 10.1371/journal.pone.0060350 (PMC3614955; doi:10.1371/journal.pone.0060350)
Supplement: Supporting Information S1 — (DOC) [file pone.0060350.s001.doc]

**DOTA-Functionalized Polylysine: A High Number of DOTA Chelates Positively Influences the Biodistribution of Enzymatic Conjugated Anti-Tumor Antibody chCE7agl**

Jürgen Grünberg^1§^, Simone Jeger^1§^, Dikran Sarko^2^, Patrick Dennler^1^, Kurt Zimmermann^1^, Walter Mier^2^, Roger Schibli^1*^

^1^Center for Radiopharmaceutical Sciences, Paul Scherrer Institute, Villigen, Switzerland; ^2^Department of Nuclear Medicine, University Hospital Heidelberg, Heidelberg, Germany

*Corresponding author, Roger Schibli: Email: [roger.schibli@PSI.ch](mailto:roger schibli).

^§^J. Grünberg and S. Jeger contributed equally to this work.

**LC-ESI-TOF MS of chCE7degl-(DOTA)_5_-decalysine and chCE7agl-(DOTA)_5_-decalysine**


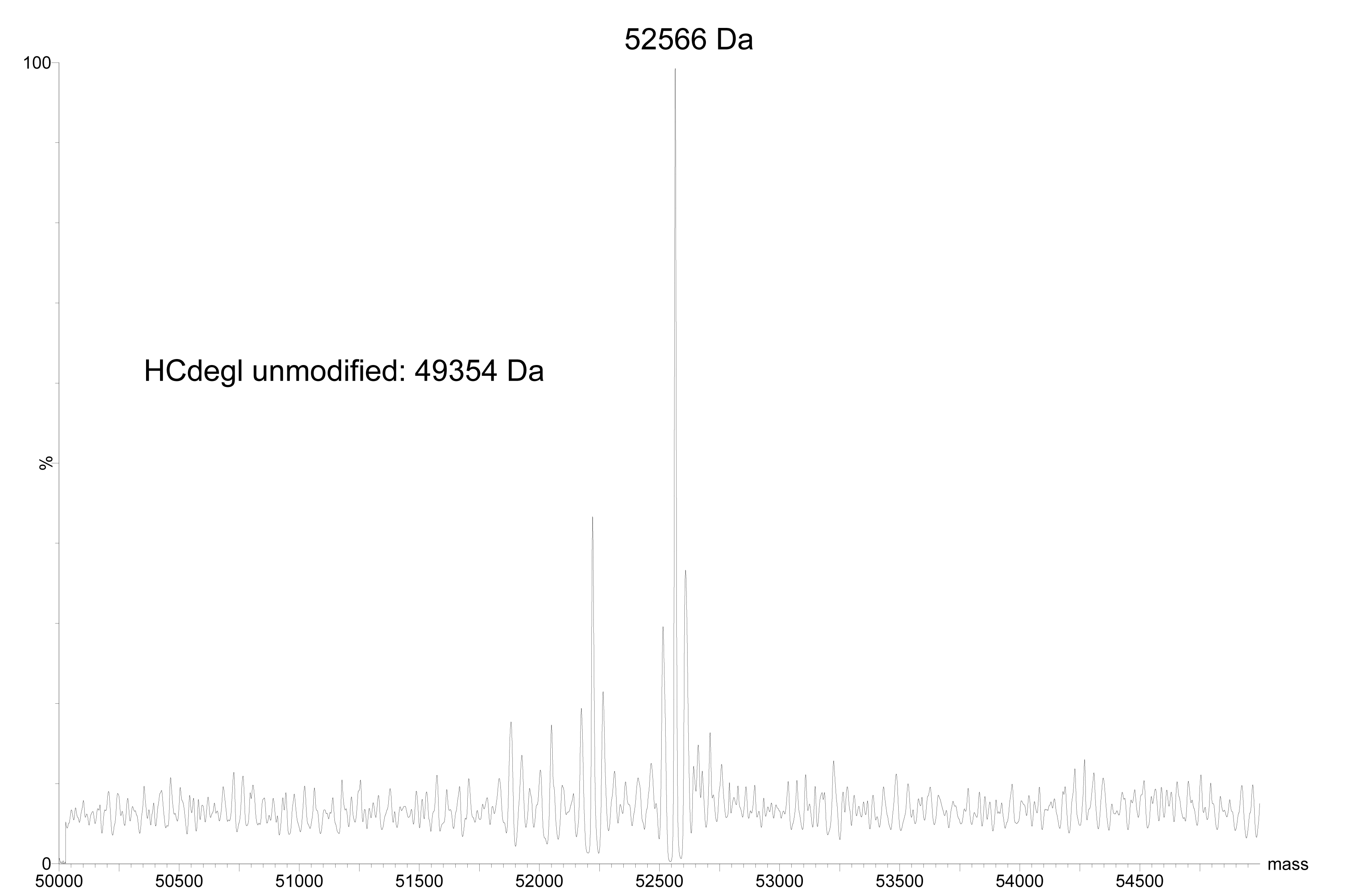


**Figure S1**: Deconvoluted mass spectrum for chCE7degl coupled with (DOTA)_5_-decalysine (MW: 3229Da)


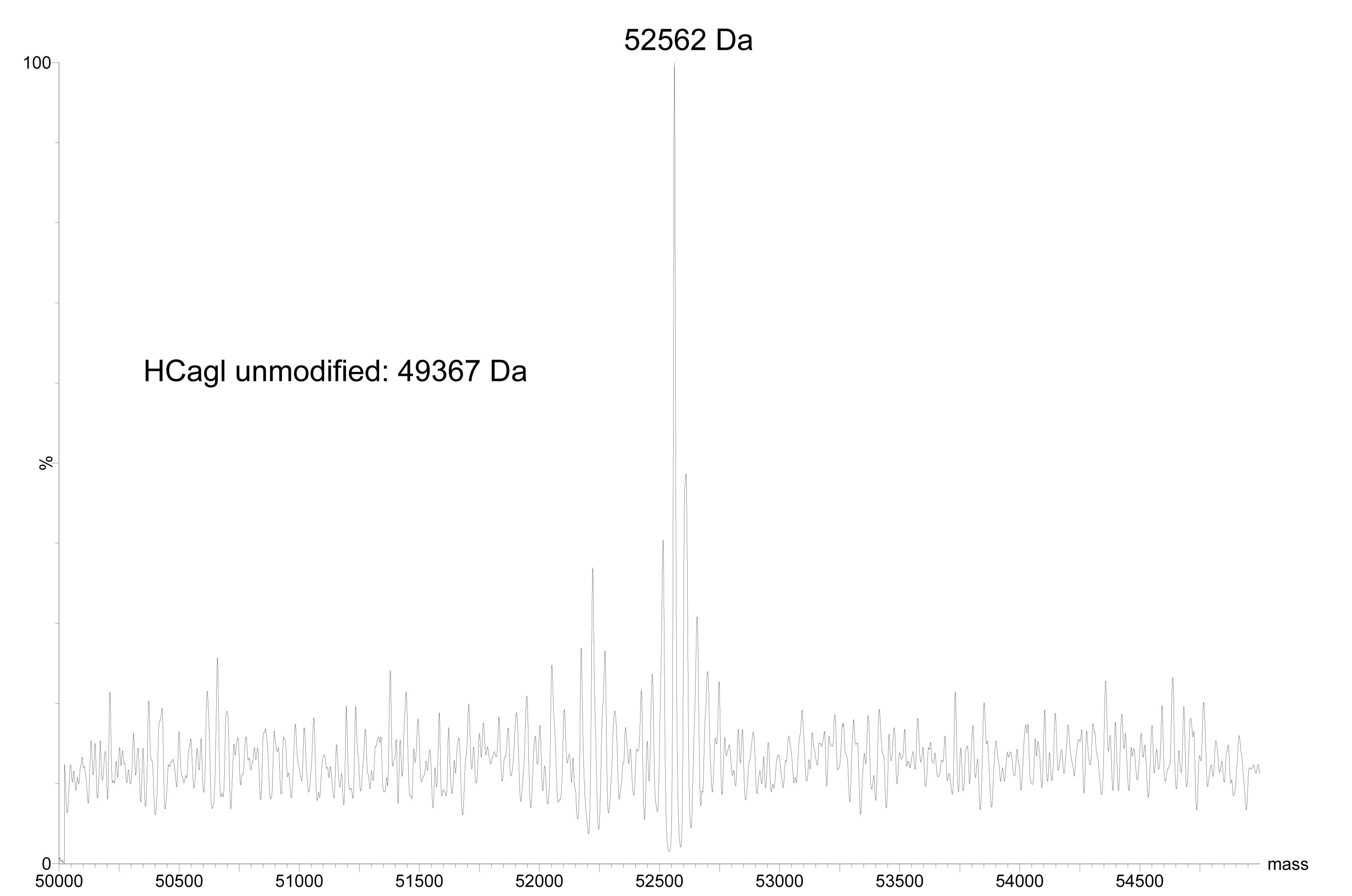


**Figure S2**: Deconvoluted mass spectrum for chCE7agl coupled with (DOTA)_5_-decalysine (MW: 3229Da)
